# Systemic treatment in breast cancer: a primer for radiologists

**DOI:** 10.1007/s13244-015-0447-4

**Published:** 2015-11-13

**Authors:** Aya Y. Michaels, Abhishek R. Keraliya, Sree Harsha Tirumani, Atul B. Shinagare, Nikhil H. Ramaiya

**Affiliations:** Department of Radiology, Brigham and Women’s Hospital, Harvard Medical School, 75 Francis Street, Boston, MA 02115 USA; Department of Imaging, Dana Farber Cancer Institute, Harvard Medical School, 450 Brookline Avenue, Boston, MA 02215 USA

**Keywords:** Breast cancer, Hormonal therapy, Molecular-targeted therapy, CT, MRI

## Abstract

**Abstract:**

Cytotoxic chemotherapy, hormonal therapy and molecular targeted therapy are the three major classes of drugs used to treat breast cancer. Imaging modalities such as computed tomography (CT), magnetic resonance imaging (MRI), ^18^F-FDG positron emission tomography (PET)/CT and bone scintigraphy each have a distinct role in monitoring response and detecting drug toxicities associated with these treatments. The purpose of this article is to elucidate the various systemic therapies used in breast cancer, with an emphasis on the role of imaging in assessing treatment response and detecting treatment-related toxicities.

***Teaching Points*:**

• *Cytotoxic chemotherapy is often used in combination with HER2-targeted and endocrine therapies*.

• *Endocrine and HER2-targeted therapies are recommended in hormone-receptor- and HER2-positive cases*.

• *CT is the workhorse for assessment of treatment response in breast cancer metastases*.

• *Alternate treatment response criteria can help in interpreting pseudoprogression in metastasis*.

• *Unique toxicities are associated with cytotoxic chemotherapy and with endocrine and HER2-targeted therapies*.

## Introduction

A paradigm shift has occurred over the past few decades in the systemic (non-surgical and non-radiation) treatment of breast cancer, with treatment regimens evolving from single-agent chemotherapy to those involving hormonal therapy, combination chemotherapy and molecular-targeted therapy (MTT) [1]. Much of this evolution can be attributed to a better understanding of the molecular complexity of breast cancer. Recent studies have revealed that there are several molecular phenotypes of breast cancer, based on receptor expression [2]. The major subtypes of these include luminal A, luminal B, HER2-positive and triple-negative (TN) breast cancer [2]. The risk of recurrence, disease management and outcome of these major subtypes vary significantly.

Anti-oestrogen therapy is an integral part of hormone receptor (HR)-positive cancers, and acts by inhibiting the growth of oestrogen-sensitive tumour cells [1]. In HER2/neu-positive cancer, MTTs directed to the HER2 receptor inhibit signal transduction pathways involved in oncogenesis [1]. MTTs have also changed the course of treatment in triple-negative breast cancer (TNBC). The increased use of these drugs in clinical practice warrants the attention of radiologists who must be familiar with the patterns of tumour response to systemic therapy and the complications associated with them. Accordingly, the purpose of this article is to provide a comprehensive review of the various cytotoxic, hormonal and biologic agents used in breast cancer in neoadjuvant, adjuvant and metastatic settings, with an emphasis on the role of imaging in assessing treatment response and drug toxicities.

## Systemic treatment in breast cancer: an overview

Systemic treatment in breast cancer includes cytotoxic chemotherapy, hormonal therapy, molecular-targeted therapy or a combination of these (Table 1). Temporally, systemic treatment can be stratified into neoadjuvant, adjuvant and metastatic settings, with each having a distinct goal [1]. The objective of neoadjuvant treatment is to downgrade the tumour in order to facilitate breast-conserving surgery and to guide post-operative chemotherapy. Adjuvant therapy is now standard practice for reducing both systemic and local recurrence, with most guidelines recommending systemic treatment for node-positive disease and tumours larger than 1 cm, irrespective of other tumour characteristics.

**Table 1:** Systemic treatment in breast cancer

Drugs	Mechanism of action	Side effects
Chemotherapy		
Anthracycline (doxorubicin and epirubicin)	Inhibits DNA and RNA synthesis	Cardiotoxicity, typhlitis, bone marrow suppression
Taxanes (paclitaxel and docetaxel)	Inhibits mitosis by stabilization of microtubule polymer	Fluid retention, neutropenic enterocolitis and typhlitis, drug-associated pneumonitis
Cyclophosphamide	Interferes with DNA replication by forming intrastrand and interstrand DNA cross-links	Hemorrhagic cystitis, Drug associated pneumonitis, diarrhoea
Capecitabine	Irreversibly inhibits thymidylate synthase	Neurotoxicity, mucositis, hand-foot syndrome
Eribulin	Inhibits mitosis by interfering with growth of microtubule	Neutropenia, diarrhoea, anaemia, peripheral nHER2athy
Hormonal therapy		
Tamoxifen	Selectively blocks estrogen receptor blockage	Hepatic steatosis and hepatotoxicity, hypercoagulability, endometrial proliferative changes
Aromatase inhibitors (anastrozole, letrozole, and exemestane)	Blocks estrogen production	Osteoporosis, arthralgia
Fulvestrant	Estrogen receptor antagonist	Elevation of liver enzymes, oedema
Molecular-targeted therapy		
Trastuzumab, pertuzumab	Interferes with the HER2/neu receptor	Cardiotoxicity, pulmonary toxicity
Lapatinib	Interrupts the HER2/neu and epidermal growth factor receptor (EGFR) pathways	Rash, diarrhoea, liver dysfunction,
Bevacizumab	Inhibits vascular endothelial growth factor (VEGF)	Hepatic steatosis, pancreatitis, cholecystitis, infection
Trastuzumab emtansine	Antibody-drug conjugate binds to HER2 receptors and enters the cell and releases the cytotoxic agent emtansine	Hepatotoxicity, thrombocytopenia

The commonly used cytotoxic drugs are taxanes (paclitaxel and docetaxel), cyclophosphamide, eribulin, capecitabine, cisplatin and anthracyclines (doxorubicin and epirubicin). Taxanes and anthracyclines are the mainstay in first-line treatment of breast cancer in the adjuvant, neoadjuvant and metastatic settings, and are often used with HER2-targeted therapies in HER2-positive breast cancer and with hormonal therapy in HR-positive breast cancer. Anthracycline-based regimens have shown greater efficacy than non-anthracycline based regimens in HER2-positive tumours [1]. National Comprehensive Cancer Network (NCCN) guidelines recommend adjuvant cytotoxic chemotherapy for all tumours greater than 1 cm, and consideration for use in tumours greater than 0.6 cm or in instances of microinvasion, regardless of molecular characteristics [1]. The drugs used in adjuvant therapy can also be used in a neoadjuvant setting, while single-agent therapy is preferred for metastatic disease in order to reduce toxicity in aggressive regimens.

Hormonal therapy is recommended for most women with HR-positive breast cancer. Tamoxifen, a selective oestrogen receptor modulator (SERM), acts as an antagonist of the oestrogen receptor (ER) in breast tissue and as agonist in the uterine endometrium [3]. Recently, another class of hormonal agents, called aromatase inhibitors (AIs), has shown superior efficacy to tamoxifen in the metastatic, neoadjuvant and adjuvant settings in postmenopausal women. Anastrozole, letrozole and exemestane are the three most widely used of these drugs. These agents work by reducing the aromatization of peripheral androgens into oestrogen [4]. Neoadjuvant hormonal therapy has shown an equivalent to superior response compared to neoadjuvant chemotherapy in HR-positive breast cancer. Studies have demonstrated greater rates of breast-conserving surgery in postmenopausal oestrogen receptor-positive breast cancer patients on neoadjuvant hormonal therapy versus neoadjuvant chemotherapy [5]. Furthermore, higher rates of breast-conserving surgery have been demonstrated in patients treated with AIs versus tamoxifen [6]. Endocrine therapy has a better toxicity profile and is preferred when chemotherapy cannot be tolerated. Another anti-oestrogen agent, fulvestrant, is an ER antagonist, and unlike tamoxifen, has no agonist effects. Fulvestrant was approved for second-line use in postmenopausal women with HR-positive metastatic breast cancer with disease progression following treatment with an anti-oestrogen [7]. Luteinizing hormone-releasing hormone (LHRH) agonists (goserelin, leuprolide), progestins (megestrol acetate), androgens (fluoxymesterone) and high-dose oestrogen (ethinyl estradiol) are other hormonal therapies used in the adjuvant and metastatic setting in breast cancer [1].

Trastuzumab (Herceptin) is a monoclonal antibody that targets cancer cells that over-express a protein called HER2 that is involved in cell growth, differentiation and blood vessel formation (angiogenesis). Up to 20 % of women with breast cancer have tumours with high levels of HER2, and many trials have demonstrated better disease-free survival with the addition of trastuzumab in combination with standard first-line chemotherapy for the treatment of HER2-positive breast cancer [8, 9]. Pertuzumab is another HER2 receptor inhibitor (HER2 dimerization inhibitor) that has been approved by the United States Food and Drug Administration (FDA) for use in metastatic breast cancer. Other targeted agents currently being studied in breast cancer include other HER2-directed agents (lapatinib); HER3 agents, which inhibit dimerization of the HER2 receptor and thus deactivate the tyrosine kinase pathway; vascular endothelial growth factor (VEGF) inhibitors (bevacizumab); poly (ADP-ribose) polymerase (PARP) inhibitors; and inhibitors of the mammalian target of rapamycin (mTOR) signalling pathway [10, 11]. Lapatinib is an orally active dual tyrosine kinase inhibitor that affects both HER2/neu and epidermal growth factor receptor (EGFR) pathways. It is being evaluated in patients with refractory CNS metastases from HER2-positive metastatic breast cancer, given its ability to achieve therapeutic levels in cerebrospinal fluid [12]. Studies such as the phase III CLEOPATRA [Clinical Evaluation Of Pertuzumab and Trastuzumab] trial have demonstrated a benefit in progression-free survival with the use of a dual anti-HER2 blockade in the metastatic setting over a single anti-HER2 agent [13]. The NCCN guidelines now recommend trastuzumab and pertuzumab (in combination) in HER2-positive metastatic disease in combination with a taxane [1].

## Role of imaging in the assessment of treatment response

Imaging plays a key role in monitoring response to treatment (Table 2). According to the NCCN guidelines, assessment of disease is most accurate when an abnormal finding is serially followed with the same imaging modality. There is no general consensus on the optimal frequency of restaging scans. Computed tomography (CT) is generally recommended every 2–4 cycles in patients on chemotherapy and every 2–6 months in patients on endocrine therapy [1]. However, if the patient develops new or worsening signs and symptoms, appropriate imaging should be pursued immediately. Patients enrolled in clinical trials tend to be restaged more frequently to determine drug efficacy.

**Table 2:** Imaging of metastatic breast cancer

Location	Imaging modalities	Remarks
CNS	MRI	• Modality of choice due to superior soft tissue resolution • DWI and perfusion MRI: help to differentiate between tumour recurrence and post-radiation changes
	PET/CT	• Differentiation between tumour recurrence and post-radiation changes
Lung	X-ray	• Initial screening modality • Less sensitive than CT for detection of pulmonary metastasis
	CT	• Modality of choice for detection of pulmonary metastasis and mediastinal adenopathy • Evaluation of treatment response as well as detecting various pulmonary toxicities associated with radiation and systemic chemotherapy
Liver	CT	• Multiphasic CT with non contrast, arterial and venous phase imaging is most commonly used for evaluation of treatment response in patients undergoing systemic therapies • Non-contrast images are useful for better delineation of lesions
	MRI	• Better characterization of suspicious lesion on CT • Hepatocyte-specific contrast agents: for detection of smaller lesions (<1 cm) and differentiation between metastasis and FNH • Better sensitivity than CT in presence of hepatic steatosis
Bone	X-ray	• Usually the first modality in the case of musculoskeletal signs and symptoms
	CT	• Assessment of axial skeleton during follow-up CT studies in patients undergoing systemic therapies
	MRI	• Superior contrast resolution and useful for evaluation of extraosseous soft tissue extension
	Tc 99m MDP bone scintigraphy	• Evaluation of asymptomatic patients to detect occult bone metastases
	PET/CT	• More sensitive than scintigraphy for detecting lytic metastases and marrow involvement • Differentiation of flare phenomena vs. true disease worsening

*MRI* magnetic resonance imaging, *PET* positron emission tomography, *CT* computed tomography, *MDP* methylene diphosphonate, *FNH* focal nodular hyperplasia

### Multidetector computed tomography (MDCT)

**Fig. 1:**
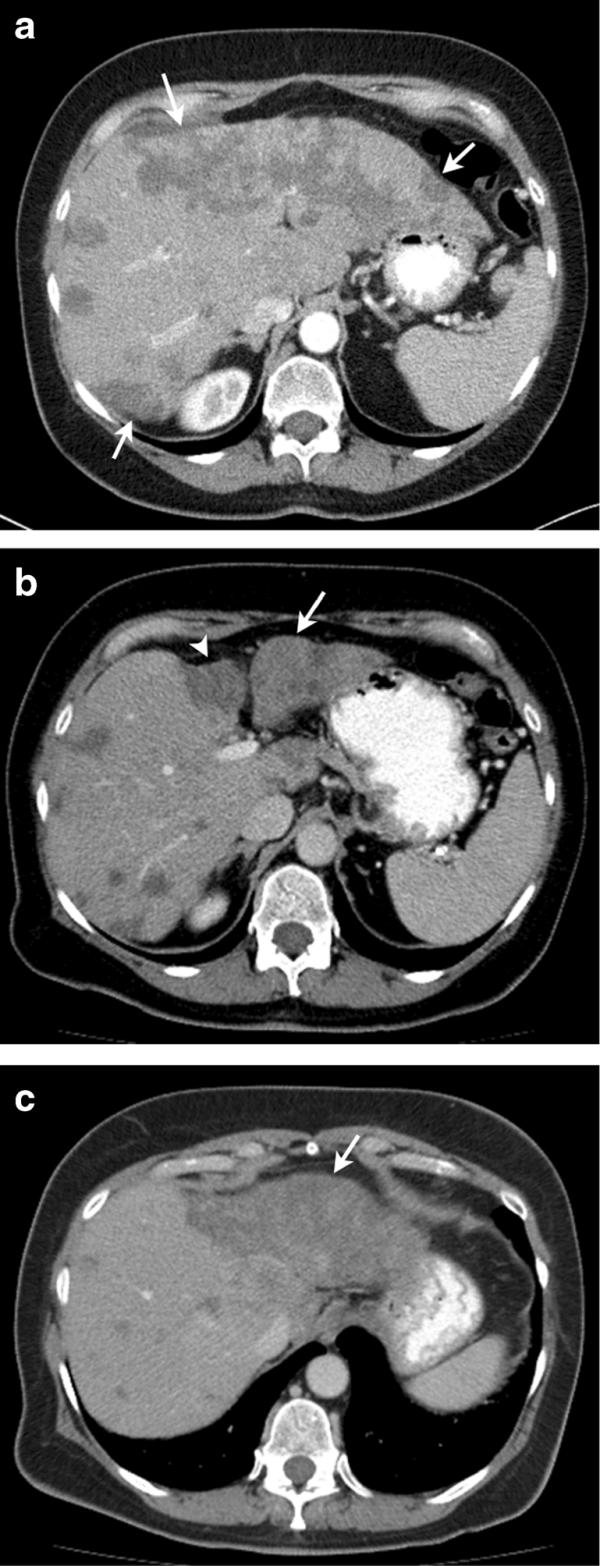
65-year-old woman with triple-positive breast cancer with multiple hepatic metastases treated with systemic chemotherapy. **a** Axial contrast-enhanced CT image before start of chemotherapy shows multiple low-attenuation hepatic metastatic lesions (*arrows*), more prominent in left lobe. **b** Axial contrast-enhanced CT image after 4 months of chemotherapy shows volume loss and surface nodularity in left lobe of liver (*arrow)*, with capsular retraction in segment IV (*arrowhead*) and decrease in size of metastatic lesions. **c** Axial contrast-enhanced CT image after 10 months of chemotherapy shows marked atrophy of left lobe of liver (*arrow*), with further decrease in size of metastatic lesions in right lobe

MDCT is the workhorse for monitoring metastatic breast cancer. Liver metastases occur in more than 50 % patients with breast cancer, and are seen as hypodense lesions. Restaging MDCT scans following systemic treatment typically show a reduction in size of the hypodense lesions in patients responding to treatment (Fig. 1). Non contrast images are useful in finding accurate tumour volume on follow-up MDCT [14]. At our institute, we routinely perform non-contrast imaging of the abdomen in all breast cancer patients, as breast cancer metastases can sometimes remain occult on the portal venous phase and can be better followed on non-contrast images on restaging scans [14]. The use of multiphasic CT with arterial and venous phase imaging has been shown to increase the detection rate of hypervascular liver metastasis, including those from breast, renal, and thyroid cancers, carcinoid tumours and melanoma [15]. There have been no large prospective studies analyzing the diagnostic accuracy of MDCT in assessing treatment response in liver metastasis from breast cancer. The Response Evaluation Criteria in Solid Tumours (RECIST) is the most widely accepted set of objective treatment response criteria [16, 17]. A recent retrospective study by He et al., however, showed that RECIST was inadequate for assessing response to targeted therapies in breast cancer liver metastasis. In their study of 39 patients with 68 liver lesions, while treatment with cytotoxic chemotherapy showed a decrease in both size and density of liver metastases, treatment with targeted therapies alone did not, although 2-year survival was better [18].

**Fig. 2:**
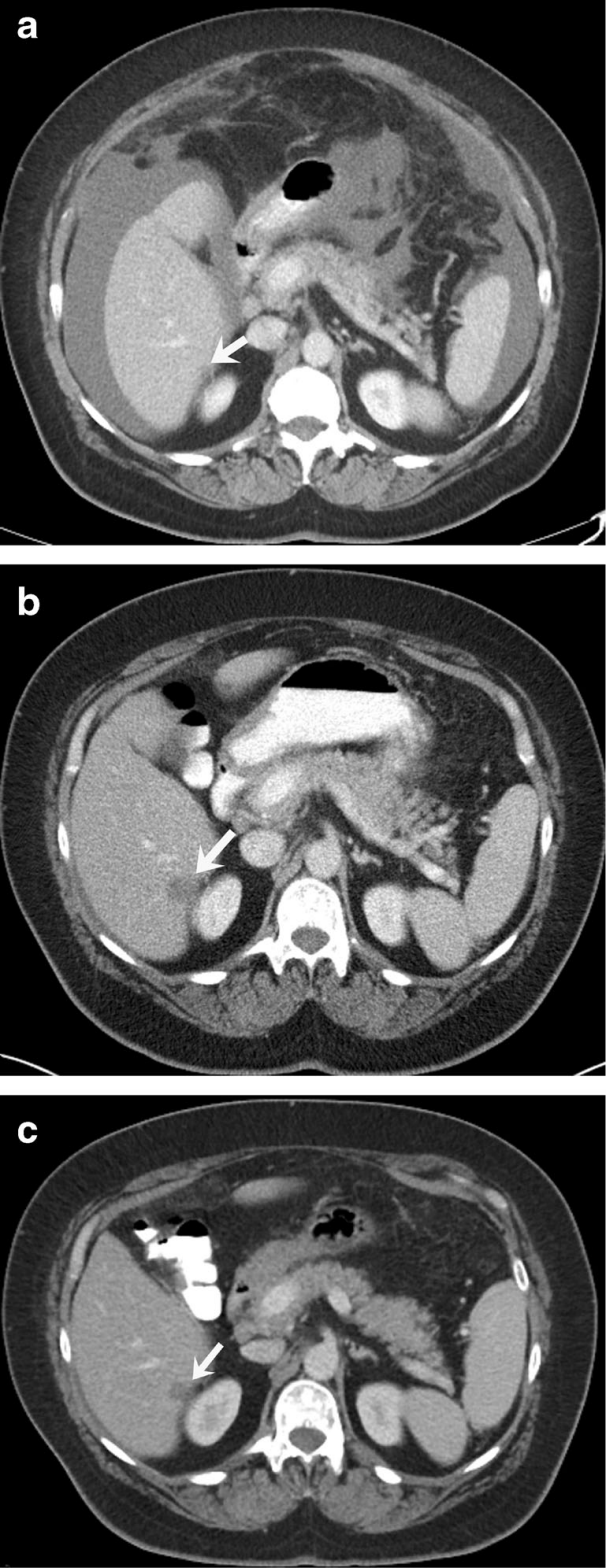
58-year-old woman with estrogen receptor-positive metastatic breast cancer treated with trastuzumab. **a** Axial contrast-enhanced CT image of the abdomen demonstrates a subcentimeter hypodense lesion in the liver (*arrow*) and ascites. **b** Follow-up scan after 3 months of treatment shows increase in the size of the liver metastasis with concurrent decrease in the ascites. Patient continued therapy due to decrease in tumour markers. **c** Repeat scan 2 months later shows decreased size of liver metastasis. The transient increase in the size of the metastasis on the interim scan (**b**) was due to decreased enhancement of the lesion suggestive of pseudoprogression

**Fig. 3:**
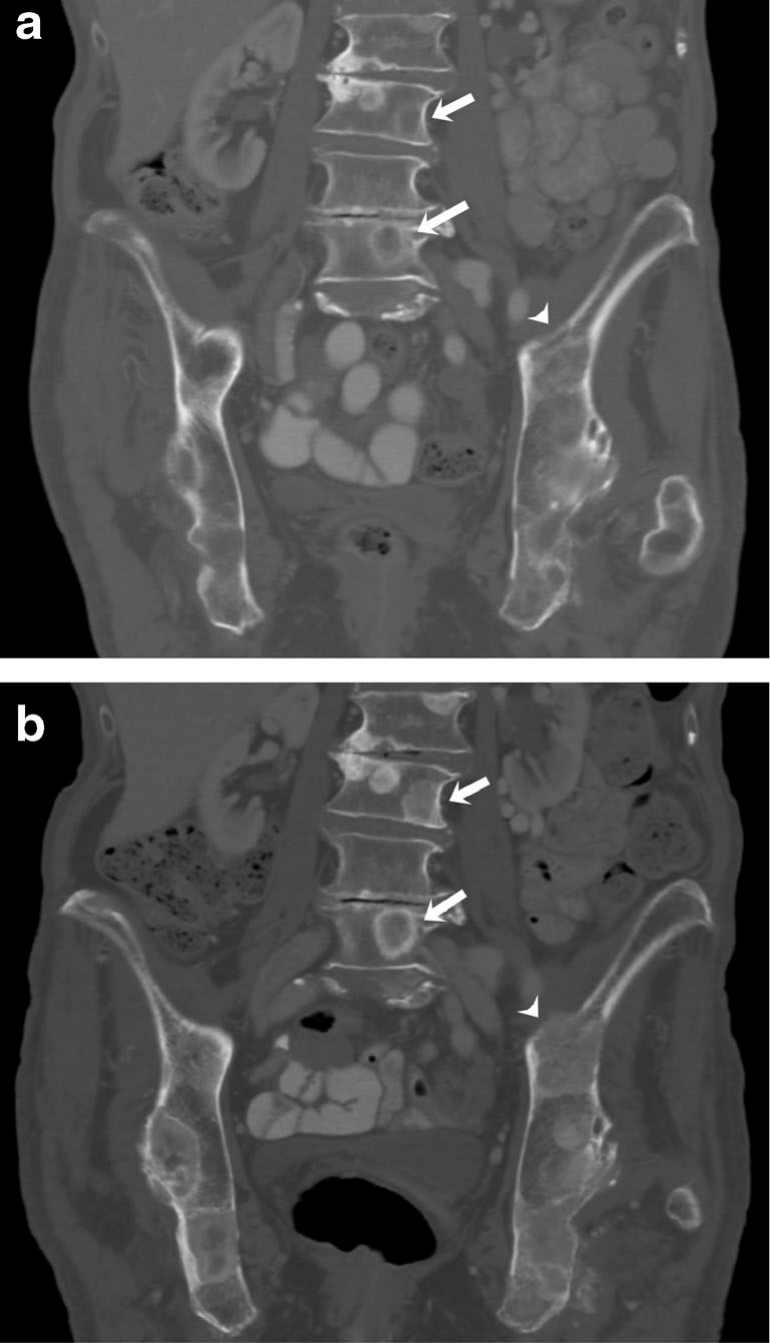
82-year-old woman with estrogen and progesterone receptor-positive breast cancer with bony metastases being treated with systemic chemotherapy. **a** Coronal pretreatment CT image of the lumbar spine and pelvis in bone window settings shows a predominantly lytic lesion involving L3 and L5 vertebrae (*arrows*) and left iliac bone (*arrowhead*). **b** Coronal CT image of the lumbar spine and pelvis after 4 months of therapy shows progressive sclerotic changes involving L3 and L5 vertebrae (*arrow*) and left iliac (*arrowhead*) lesions representative of treatment effect. This response is in agreement with patient’s improved clinical status and tumour marker levels

A classic finding seen in treated breast cancer metastases is hepatic capsular retraction, or “pseudocirrhosis” (Fig. 1). In patients undergoing chemotherapy, pseudocirrhosis has been documented as a form of treatment response, described as capsular retraction secondary to shrinkage and retraction of tumours. This finding occurs more often in larger than smaller hepatic lesions, suggesting that intrinsic pathologic characteristics such as tumour growth or fibrosis, rather than tumour response alone, may contribute to capsular retraction [19]. Treatment with molecular-targeted drugs can reduce the enhancement of hepatic metastatic lesions compared to adjacent liver parenchyma. This phenomenon, known as pseudoprogression, with the appearance of apparent new lesions or transient enlargement of existing lesions is frequently seen on post-treatment follow-up CT examinations (Fig. 2) [20]. Similarly, lytic osseous metastatic lesions show osteoblastic response after systemic therapy. Restaging scans may show apparent new sclerotic lesions due to osteoblastic treatment response, which can be confused as new bone metastases (pseudoprogression) (Fig. 3) [21, 22]. Clinical and biochemical correlation (tumours markers) helps to assess treatment response in such cases.

Thoracic metastases in breast cancer can involve pulmonary parenchyma, airways, pleura and thoracic lymph nodes [23]. Imaging patterns of lung parenchymal metastases from breast cancer include solitary or multiple lung nodules, endobronchial nodules, lymphangitic carcinomatosis and air–space consolidation. Pleural disease from breast cancer usually manifests as pleural nodularity, thickening and effusions. Metastatic parenchymal nodules are generally solid, spheric or ovoid in shape, sharply marginated, and located mostly in the periphery of the lung in contrast to other aetiologies such as infection or inflammation [23]. However, a solitary pulmonary nodule appearing in a patient with breast cancer is not always suggestive of metastatic disease, as more than 50 % of the nodules may have aetiologies such as primary lung tumour or other benign lesions, and histological confirmation is necessary [24]. Pulmonary nodules respond to systemic treatment with a reduction in size. Cavitation of pulmonary nodules is uncommonly seen as secondary to both cytotoxic chemotherapy and newer anti-angiogenic agents. To prevent misclassification as disease progression, alternative methods of measuring cavitary lesions (i.e. exclusion of the air component during measurement) have been described in the primary lung cancer literature [25].

### Magnetic resonance Imaging (MRI)

#### Breast MRI

**Fig. 4:**
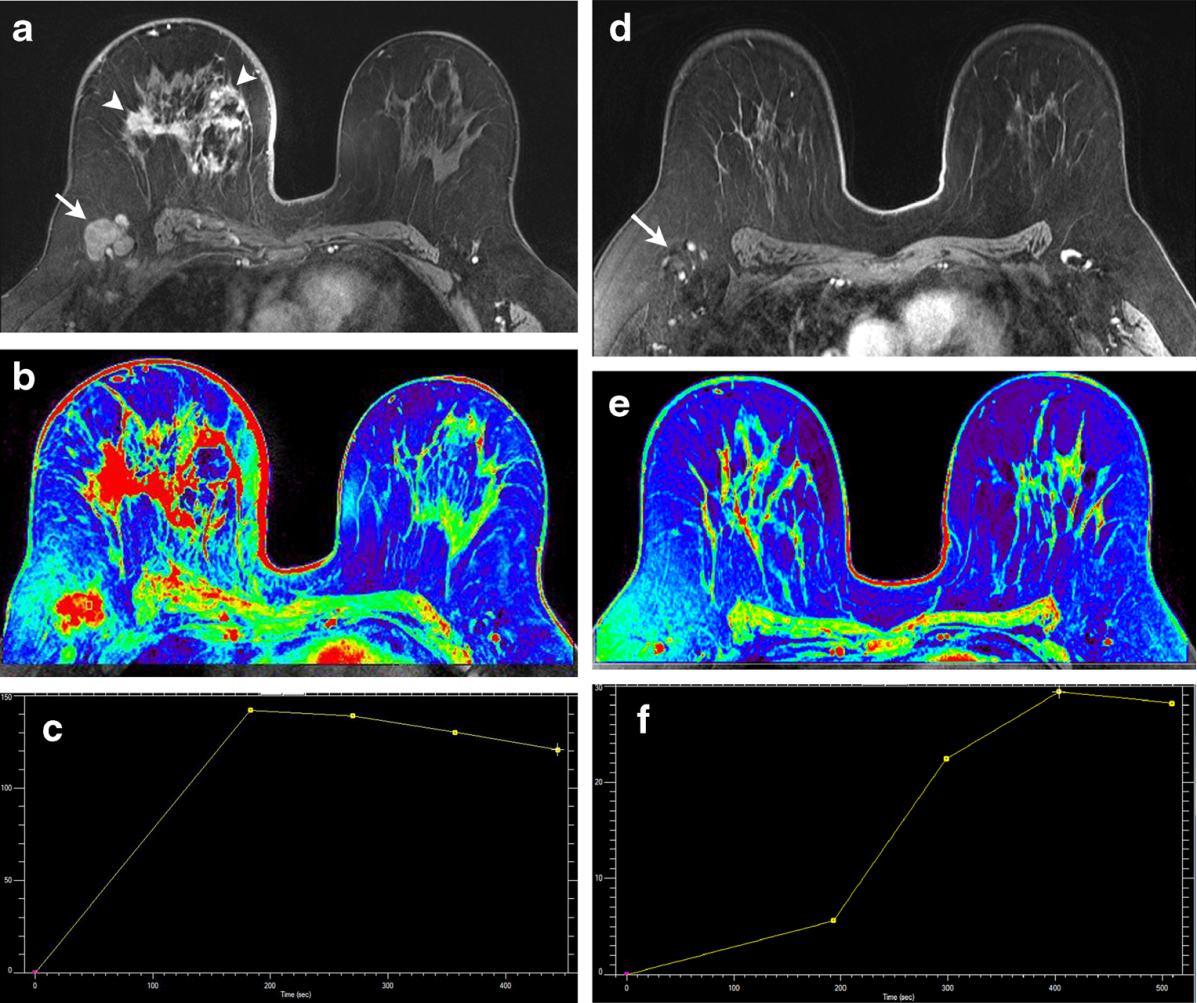
36-year-old woman with invasive lobular carcinoma in the right breast. **a** Axial contrast-enhanced T1-weighted MR image before start of neoadjuvant chemotherapy shows diffuse skin thickening and edema involving the right breast, with multiple enhancing masses (*arrowheads*) and enlarged right axillary lymph nodes (*arrow*). **b**, **c** Corresponding color map and enhancement kinetic curve demonstrate intense enhancement and washout consistent with malignancy. **d** Axial contrast-enhanced T1-weighted MR image after 4 months of therapy shows interval resolution of skin thickening and breast masses, with marked decrease in size of axillary lymph nodes (*arrow*). **e**, **f** Corresponding color map and enhancement curve demonstrate nearly complete resolution of the breast mass and axillary modes

In the neoadjuvant setting, breast magnetic resonance (MR) imaging is a useful tool for monitoring response to chemotherapy, and is superior to clinical examination, ultrasound and mammography [26]. The tumour size correlation coefficient between MRI and pathologic analysis is very high (more than 0.9) compared to that of clinical examination and mammography (around 0.7) [26]. MRI has demonstrated high sensitivity (96 %) and accuracy (89 %) for the detection of residual disease after neoadjuvant chemotherapy [27]. Various studies have shown that the accuracy of MRI in this setting varies with the molecular subtype of breast cancer, the pattern of enhancement on pre-treatment MRI and the nuclear grade [27]. MRI has demonstrated higher accuracy in assessing treatment response and size changes in tumours with HER2+ or triple-negative status, and lower accuracy with luminal-type breast cancer, low nuclear grade and diffuse non-mass-like enhancement on initial MRI [27, 28]. Dynamic contrast-enhanced MRI, in addition to morphological analysis, offers the advantage of evaluating enhancement kinetics, which correlate with angiogenic changes in response to neoadjuvant therapy (Fig. 4) [29].

#### Liver MRI

**Fig. 5:**
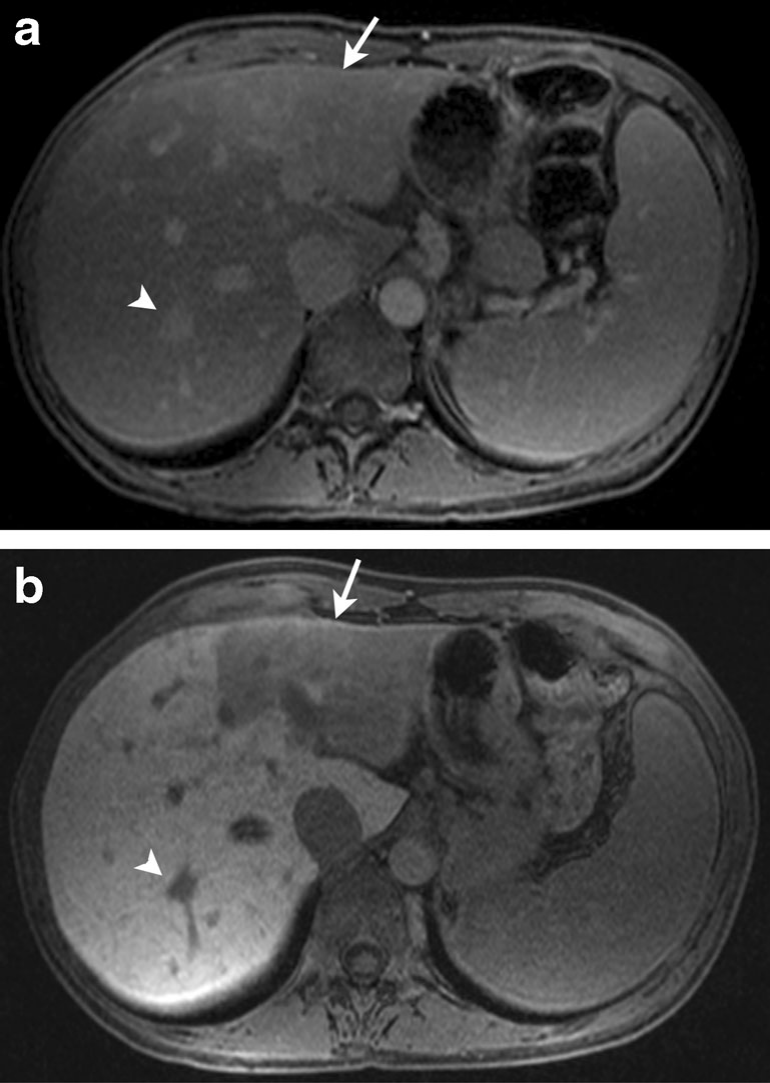
46-year-old woman with metastatic hormone receptor-positive breast cancer. **a** Axial fat-suppressed T1-weighted MR image in venous phase after administration of intravenous gadopentetate dimeglumine (Magnevist) shows ill-defined hyperenhancement in segment III of left lobe of liver (*arrow*) and focal hyper-enhancing lesion in segment VII of right lobe of liver. **b** Axial fat-suppressed T1-weighted MR image in the hepatocyte phase (20-min delay) after administration of intravenous gadoxetate disodium (Eovist) increases conspicuity of the hepatic lesions in segment III (*arrow*) and segment VII (*arrowhead*)

Superior soft tissue characterization renders MR imaging particularly helpful in the evaluation of liver metastases. Dynamic contrast-enhanced MRI has greater than 90 % sensitivity in the identification of hepatic metastases, compared to contrast-enhanced CT at around 80 % [30]. Hepatocyte-specific contrast agents, including gadoxetate disodium (Eovist/Primovist; Bayer HealthCare, Leverkusen, Germany) and gadobenate dimeglumine (MultiHance; Bracco Diagnostics Inc., Princeton, NJ, USA), are excreted through a combination of biliary and renal routes [31]. Gadoxetate disodium has approximately 50 % biliary excretion in patients with normal liver and renal function, and the addition of a hepatobiliary phase in dynamic-contrast MRI has shown increased sensitivity (more than 10 %) in detecting metastatic lesions over dynamic MRI alone (Fig. 5) [32]. These agents are particularly useful in detecting small metastases, less than 1 cm in size [33], and for differentiation of metastatic lesions from focal nodular hyperplasia (FNH) [34]. In the assessment of treatment response, MRI with in-phase and out-of-phase imaging can be more effective for the evaluation of lesions in the setting of chemotherapy-induced hepatic steatosis, which can obscure liver metastases [20]. Diffusion-weighted MRI has been shown in a few studies to predict early response to treatment in breast cancer [35].

#### Brain MRI

**Fig. 6:**
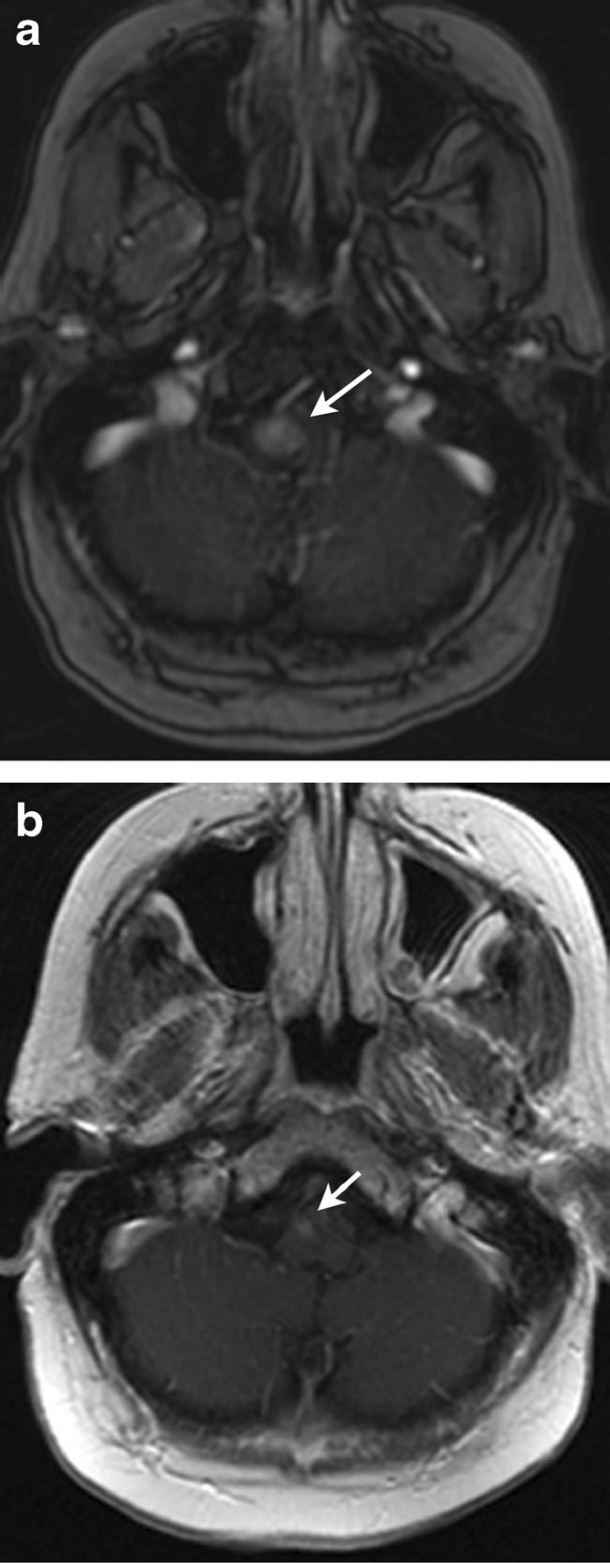
62-year-old woman with metastatic breast cancer and new complaint of numbness and tingling in her left hand and left foot. **a** Axial contrast-enhanced T1-weighted MR brain image shows homogenously enhancing lesion within the right lateral aspect of the medulla (*arrow*) suggestive of metastasis. **b** Axial contrast-enhanced T1-weighted MR brain image after whole-brain radiation and 2 months of therapy with lapatinib shows significant interval decrease in size of enhancing lesion in medulla (*arrow*)

MRI is the best modality for localizing brain metastases and assessing response to treatment (Fig. 6). In patients with a good prognosis, local control with surgery and/or radiation treatment (either stereotactic or whole-brain radiation therapy) can be pursued. Measuring the response is often difficult, as the appearance of post-treatment changes can mimic recurrent or residual pathology. Determining whether a ring-enhancing lesion at the site of the treated tumour represents treatment response (radiation necrosis or granulation tissue surrounding a resection cavity) versus recurrence can be particularly challenging. The chronology of the appearance of the lesion can be helpful, as radiation necrosis typically appears within 4 months and resolves after 2–3 years [36]. Advanced MRI techniques such as diffusion and perfusion MRI are helpful for differentiating between tumour recurrence and post-treatment changes [37]. Recurrent tumours show restricted diffusion, whereas post-treatment changes have high signal intensity on apparent diffusion coefficient (ADC) maps. Metastatic lesions on perfusion MR will typically demonstrate increased relative cerebral blood volume (rCBV), while radiation necrosis usually shows decreased rCBV [36].

### ^18^F-FDG PET/CT

**Fig. 7:**
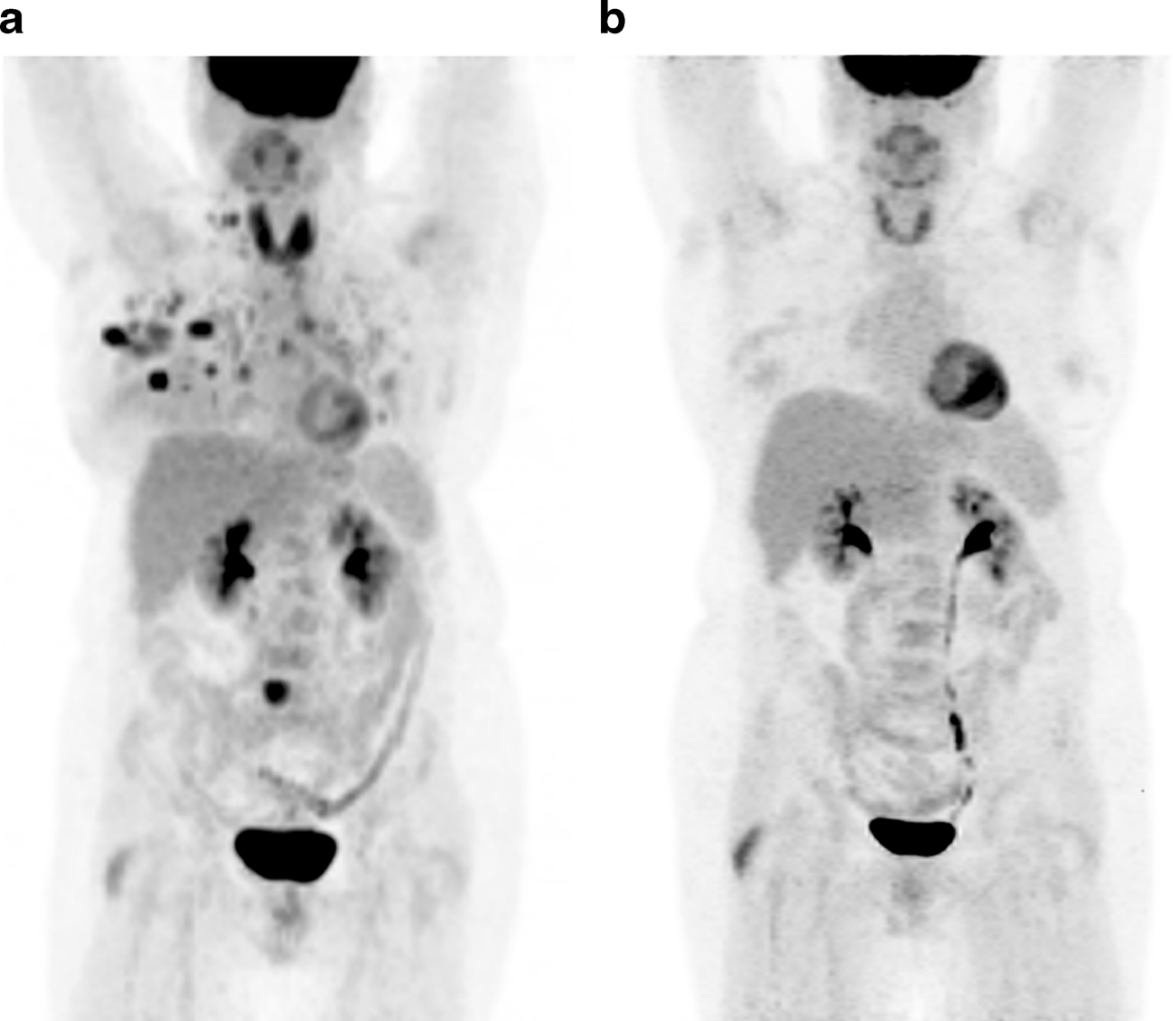
36-year-old woman with invasive lobular carcinoma in the right breast (same patient as in Fig. 4). **a** Coronal maximum-intensity projection (MIP) 18F-FDG PET image also performed prior to start of treatment shows multiple FDG-avid nodules in the right breast, with right axillary, subpectoral, cervical, mediastinal, and bilateral hilar lymphadenopathy. Also note the FDG-avid focus in the sacrum suggestive of osseous metastasis. **b** Coronal maximum-intensity projection (MIP) 18F-FDG PET image after 6 months of therapy shows significant decrease in FDG-avid lesions suggestive of response to therapy

Positron emission tomography (PET)/CT is a useful diagnostic modality in the event of equivocal findings on conventional staging techniques, especially in patients with locally advanced or metastatic disease [1]. In the neoadjuvant setting, a decrease in maximum standardized uptake value (SUV_max_) of 60 % from baseline has shown up to 96 % specificity in predicting pathologic complete response (pCR) after just one course of chemotherapy (Fig. 7) [38]. This contributes important prognostic information, as patients with pCR have significantly higher disease-free and overall survival rates than non-responders. PET/CT is also valuable for detecting metabolic changes following treatment with antineoplastic agents. Prior to PET/CT, methods for monitoring therapeutic effectiveness were limited to physical exam or conventional imaging, thus relying on physical and morphological information. PET/CT is effective for monitoring physiological response after one treatment, prior to pathologic confirmation at the time of surgery, and thus decisions regarding the continuation or modification of therapy can be made early in the treatment course [39]. The pooled sensitivity and specificity of FDG-PET/CT in predicting histopathologic response in primary breast cancer is 81 and 79 %, respectively [40]. Similar to that of MRI, the performance of FDG-PET/CT in predicting pathologic complete response is better in HER2-positive and triple-negative tumours than in the luminal subtype [40]. In the metastatic setting, the sensitivity and specificity of FDG-PET/CT is 93 and 82 %, respectively [41]. The degree of reduction in FDG uptake in metastatic lesions after the first cycle of chemotherapy has been shown in some studies to differentiate responders from non-responders [41]. In evaluation of osseous metastasis, FDG-PET/CT can help in identifying viable metastases, as treated metastases become FDG-negative but remain osteoblastic on CT [41]. In cases of brain metastasis, FDG-PET is useful for differentiating between post-radiation necrosis and tumour recurrence [42]. One study found that dual-phase FDG-PET imaging of the brain was superior to the standard single-phase FDG-PET in differentiating recurrent metastasis from post-treatment necrosis [43].

### 99mTc methylene diphosphonate (MDP) bone scintigraphy

**Fig. 8:**
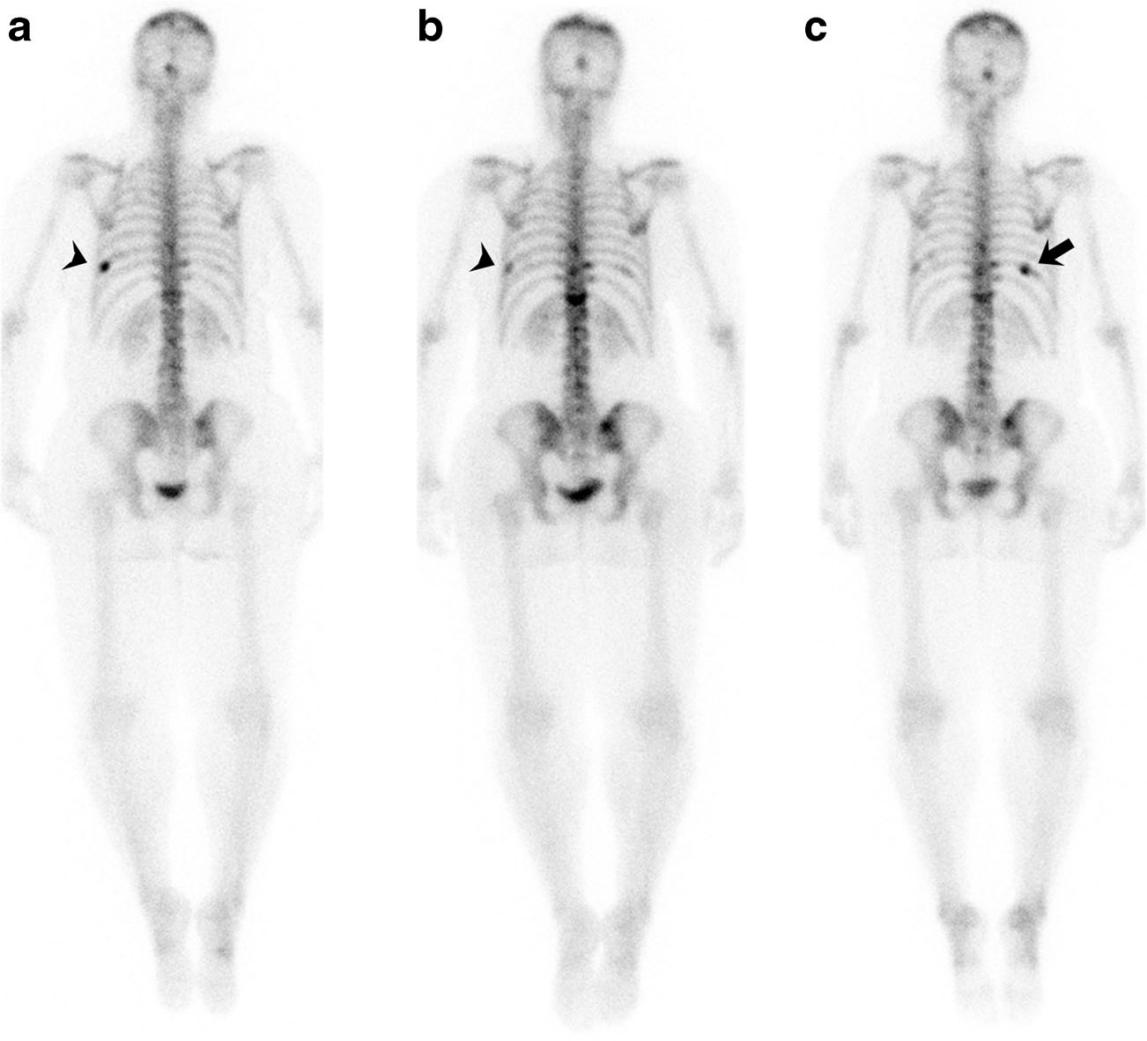
61-year-old woman with breast cancer and osseous metastases being treated with capecitabine. **a** Pretreatment bone scan shows multifocal abnormal radiotracer uptake in calvarium, multiple thoracic and lumbar vertebrae, and bilateral iliac bones. Focal uptake in left 9th rib (*arrowhead*) corresponds to recent traumatic fracture. **b** Post-treatment bone scan after 2 months of therapy shows slight increase in the intensity and extent of radiotracer uptake in the multiple known metastases, uptake in calvarium, multiple thoracic, and lumbar vertebrae and bilateral iliac bones. Given the improvement in tumour markers, this finding was regarded as response to treatment with a scintigraphic flare phenomenon. Uptake in left 9th rib is decreased (*arrowhead*). **c** Post-treatment bone scan after 4 months of therapy shows interval decrease in the intensity and extent of radiotracer uptake in the multiple known metastases. Focal uptake in right 10th rib (*arrow*) corresponds to a new traumatic fracture

Bone is the most common site of breast cancer metastasis. Bone scintigraphy is the most widely used modality for detecting bone metastases, and demonstrates osteoblastic activity as areas of increased radiotracer uptake. However, certain characteristic false-negative and false-positive findings on scintigraphy should be kept in mind in assessing treatment response. Scintigraphy sometimes demonstrates an apparent worsening of abnormalities, known as the “flare phenomenon,” characterized by increased activity in known lesions or new lesions during the first 3 months after treatment as a result of bone repair (Fig. 8) [44]. This finding should not be confused with progression of disease, and should be followed up with another bone scan in 4 to 6 months. A finding that persists beyond 6 months, however, is more likely indicative of disease progression. The apparent worsening or new sclerotic osseous lesions should be interpreted with caution, and should not be considered new or progressive disease, particularly in the setting of improving tumour markers and clinical status and the absence of other bone progression. The concept of increased osteoblastic healing reaction was introduced as a criterion for treatment response by the M.D. Anderson Cancer Center in 2004 [45]. In the revised criteria, CT and MRI are included as imaging modalities for assessing treatment response in bony metastases. The appearance of peripheral sclerosis around initial lytic lesions or sclerosis of previously undetectable lesions on CT or radiographs is considered a partial response. The rapid regression of lesions on scintigraphy was excluded as a partial response, as this finding may represent progression of lytic bony lesions. Bone scintigraphy is not sensitive for lytic osseous metastases given the low amount or absence of osteoblastic activity in these lesions. ^18^F-FDG PET is more sensitive than scintigraphy in detecting lytic metastases and marrow involvement [46]. The recently proposed PERCIST (PET Response Criteria in Solid Tumours) guidelines involve the role of FDG PET in treatment response, with an emphasis on change in tumour metabolism after therapy over anatomically based criteria [47].

## Complications associated with treatment: the role of imaging

### Cytotoxic agents

**Fig. 9:**
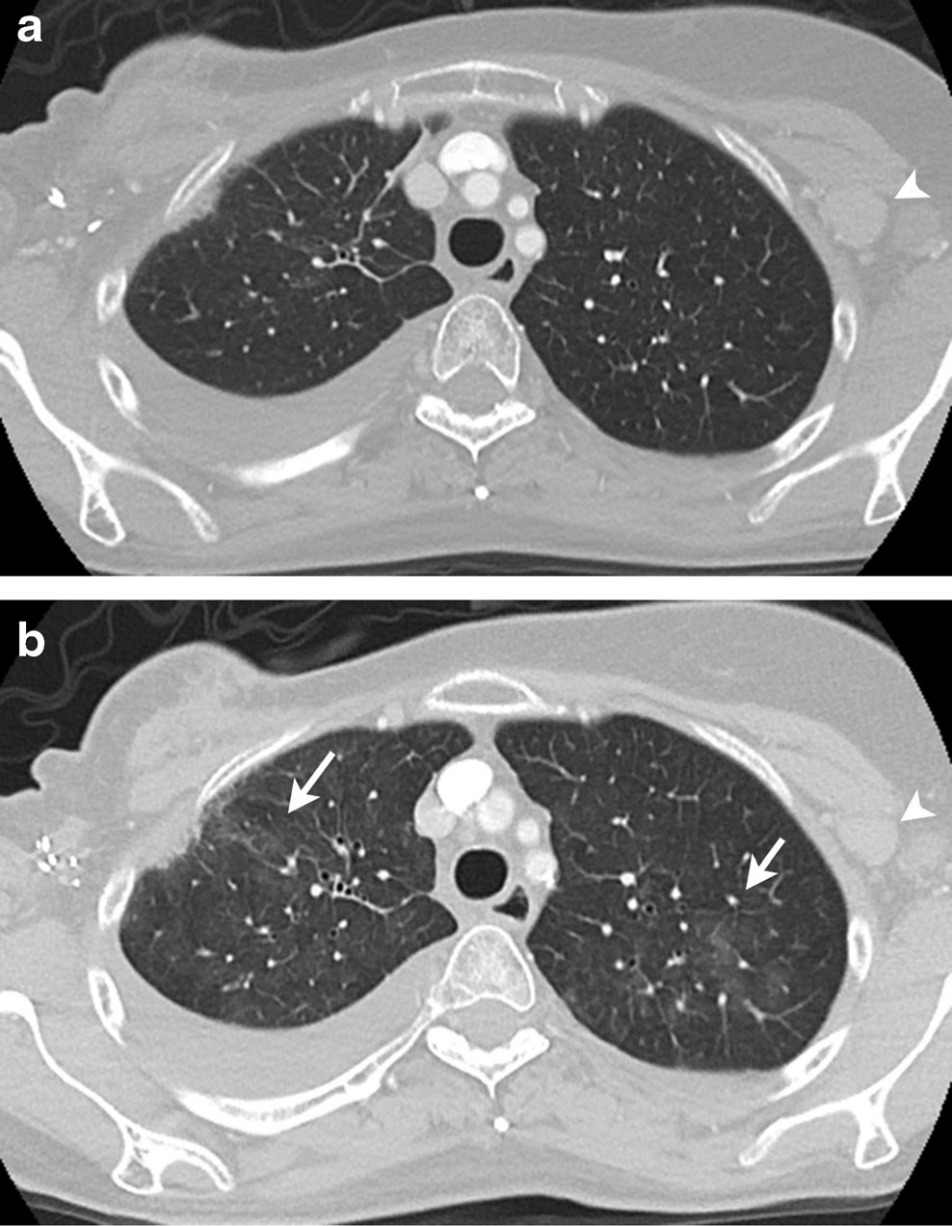
69-year-old woman with metastatic hormone receptor-positive, HER2/neu-positive breast cancer being treated with paclitaxel. Axial contrast-enhanced lung window CT image before (**a**) and after (**b**) treatment with paclitaxel shows interval appearance of patchy ground-glass opacities in upper lobes of lungs (*arrow* in **b**) suggestive of drug-associated pneumonitis. Also note the decreased size of left axillary lymph node (*arrowheads* in **a** and **b**) suggestive of treatment response

CT is also helpful in monitoring treatment-related complications. Pulmonary toxicity is a common drug-related complication seen with taxanes and methotrexate. CT findings include pulmonary infiltrates presenting in early onset as bilateral reticular, ground-glass or consolidative opacities, pulmonary oedema, and pleural effusions, and as fibrosis in late disease (Fig. 9) [48]. Pulmonary infiltrates, however, are a non-specific finding, and can also result from disease progression and infection; thus clinical factors should also be taken into consideration.

Neutropenic enterocolitis and typhlitis (neutropenic enterocolitis of the cecum) are the most common GI side effects of chemotherapy, and are associated with several cytotoxic breast cancer drugs including doxorubicin, docetaxel, paclitaxel and platinum agents. CT findings include colonic wall thickening and oedema and/or necrosis, pericolonic fat stranding, ascites, pneumatosis intestinalis, and free air in the setting of bowel necrosis and perforation. While most frequently seen in the right colon, any colonic or small bowel segment can be involved. Because rapidly dividing GI mucosal cells are also vulnerable to cytotoxic agents, GI ulceration, which may further lead to perforation, is another treatment complication most commonly associated with doxorubicin and taxanes. Pneumatosis intestinalis may be observed with cyclophosphamide, doxorubicin, cisplatin and docetaxel [49].

Fluid retention, likely due to capillary protein leakage, has been seen in patients treated with docetaxel, and may manifest as peripheral oedema and as pleural and pericardial effusions [50]. Platinum-based agents carry an increased risk of thrombus formation [51]. Hemorrhagic cystitis is a well-known complication of cyclophosphamide, manifesting radiologically as diffuse thickening and nodularity of the bladder wall secondary to urothelial toxicity. This complication usually occurs early in treatment and is preventable with hydration and concurrent treatment with mesna, which neutralizes the toxic metabolic products within the bladder [52].

### Hormones

**Fig. 10:**
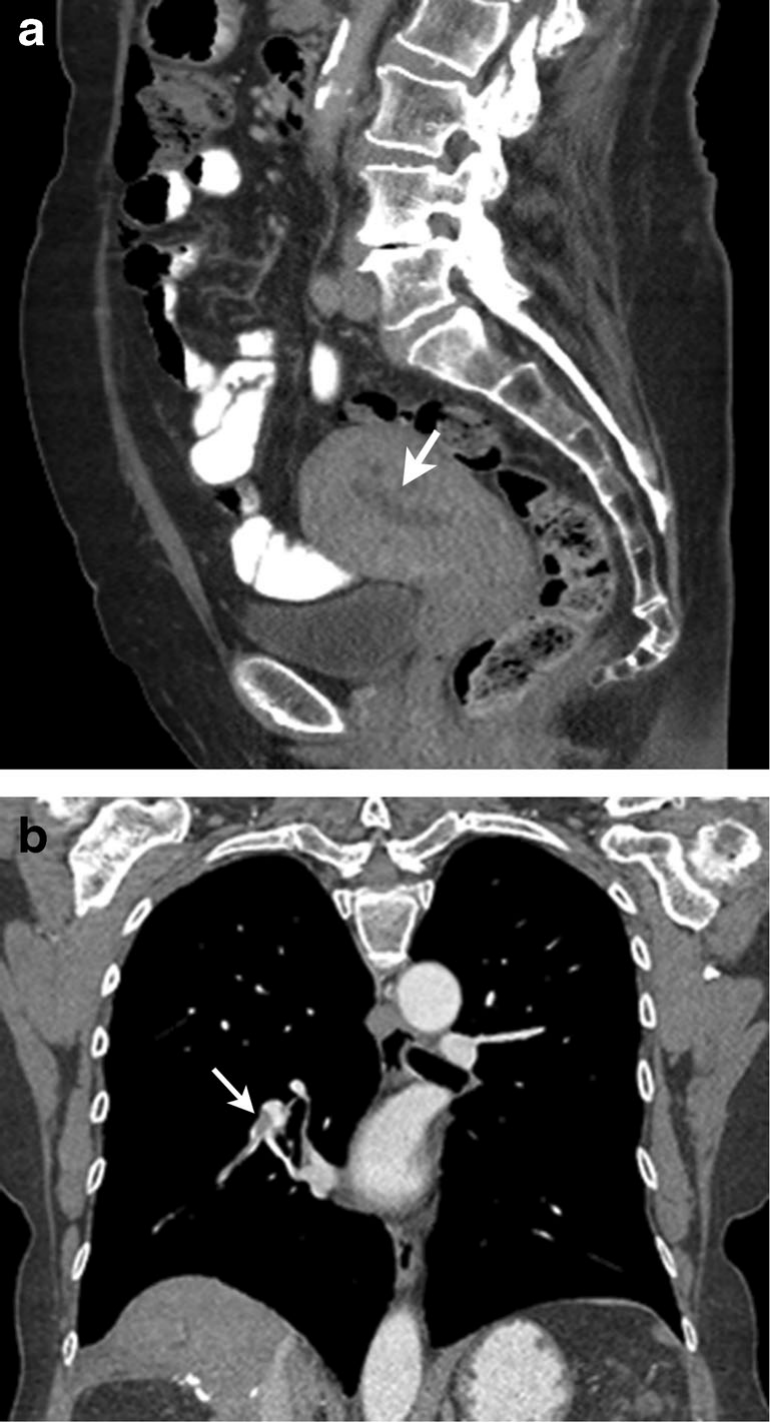
72-year-old woman with metastatic hormone receptor-positive breast cancer being treated with tamoxifen. **a** Sagittal contrast-enhanced CT image of the pelvis shows irregular thickening of the endometrial lining (*arrow*) suggestive of endometrial hyperplasia. **b** Coronal contrast-enhanced CT image of the pelvis during routine restaging study shows hypodense filling defect (*arrow*) in descending branch of right pulmonary artery suggestive of pulmonary embolism. The patient had no complaints of chest pain

Patients receiving treatment with tamoxifen have an increased prevalence of endometrial hyperplasia, endometrial polyps and endometrial carcinoma (Fig. 10). Transvaginal ultrasound (US) and hysterosonography are useful tools for endometrial assessment, which is especially helpful in women treated with tamoxifen. While the agonistic effects of tamoxifen on estrogen receptors are beneficial in some tissues—for example, in lowering serum cholesterol and protecting against bone loss and cardiovascular disease—its proliferative effects on the uterine endometrium increase the risk of endometrial cancer [53]. On transvaginal US, endometrial carcinomas are typically diffusely or partially echogenic. As many patients treated on tamoxifen have endometrial thickening and underlying adenomyosis, poorly defined endometrial thickening is not a specific sign. Hysterosonography may be of further help in identifying an irregular, inhomogeneous mass or focal thickening in the endometrium [54]. Lack of distensibility of the endometrial cavity may also suggest carcinoma. MRI is useful for the characterization of endometrial abnormalities associated with tamoxifen therapy [55].

Tamoxifen causes hypercoagulability, increasing the risk of thromboembolic phenomena and catheter-related thrombosis (Fig. 8) [56].

**Fig. 11:**
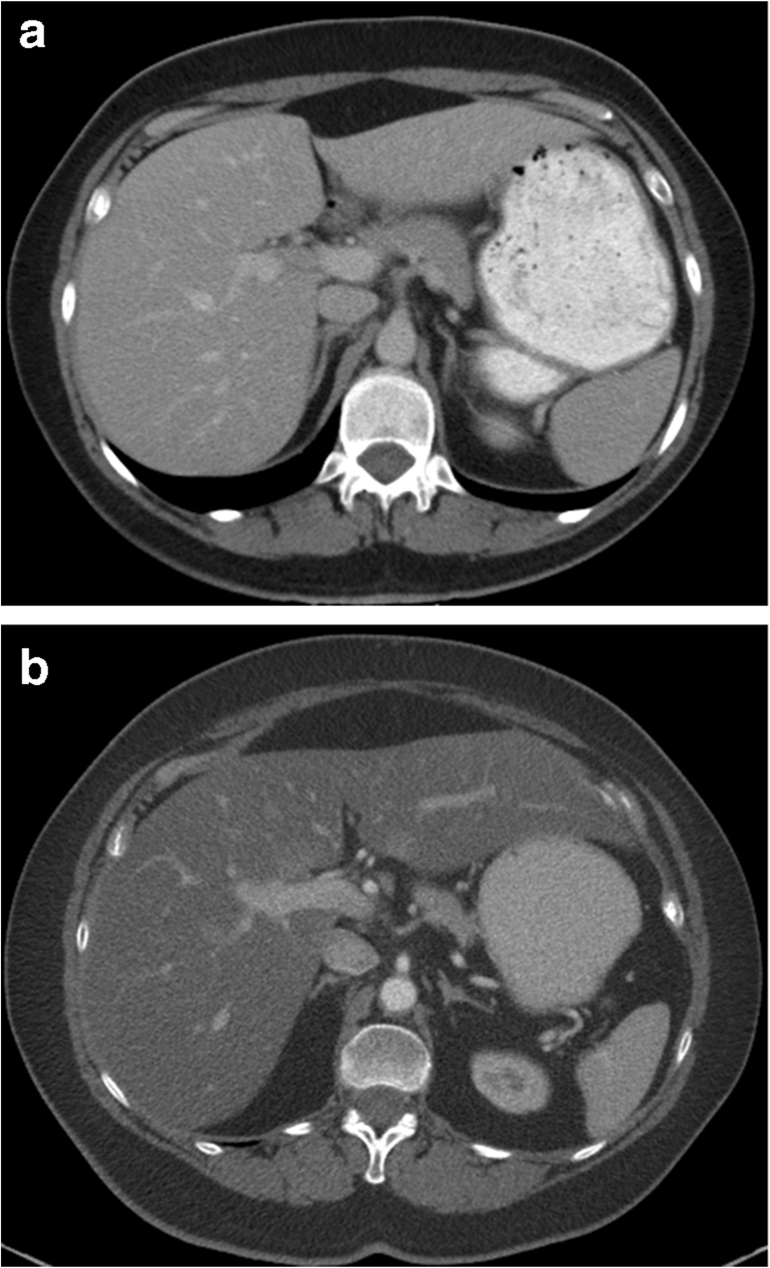
72-year-old woman with metastatic hormone receptor-positive breast cancer being treated with tamoxifen. Axial contrast-enhanced CT image of the pelvis before (**a**) and after (**b**) 6 months of treatment with tamoxifen shows diffuse hypodensity of liver parenchyma on image (**b**) compared to image (**a**) suggestive of diffuse fatty deposition

Fatty liver is seen in up to 30 % of patients treated with tamoxifen (Fig. 11), and can be present as early as 3 months after initiation of treatment and can persist for more than 4 years after discontinuation [57]. Risk factors for hepatic changes associated with tamoxifen include preexisting conditions such as diabetes, obesity and hepatic steatosis. Fatty liver may rarely progress to steatohepatitis and cirrhosis. On ultrasound, hepatic steatosis presents as an echogenic liver, while on CT, the liver appears hypodense in comparison to the spleen (Fig. 9). On CT, fatty infiltration of the liver may obscure the presence of hypodense metastases. MR is helpful, as in-phase and out-of-phase imaging will demonstrate intracellular fatty deposition with signal dropout on out-of-phase sequences [58].

In postmenopausal women, the primary source of oestrogen is through conversion from adrenal androgen by aromatase. AIs prevent the conversion of androgens to estrogens, thereby causing a relatively rapid reduction in circulating oestrogen and an acceleration of bone loss at more than twice the rate of physiologic postmenopausal loss [59]. Regular bone marrow density monitoring is thus highly recommended. Bisphosphonates are the first line of therapy for the prevention and treatment of aromatase inhibitor-associated bone loss, as women on AIs are at increased risk of fractures [59].

### Molecular-targeted therapies

**Fig. 12:**
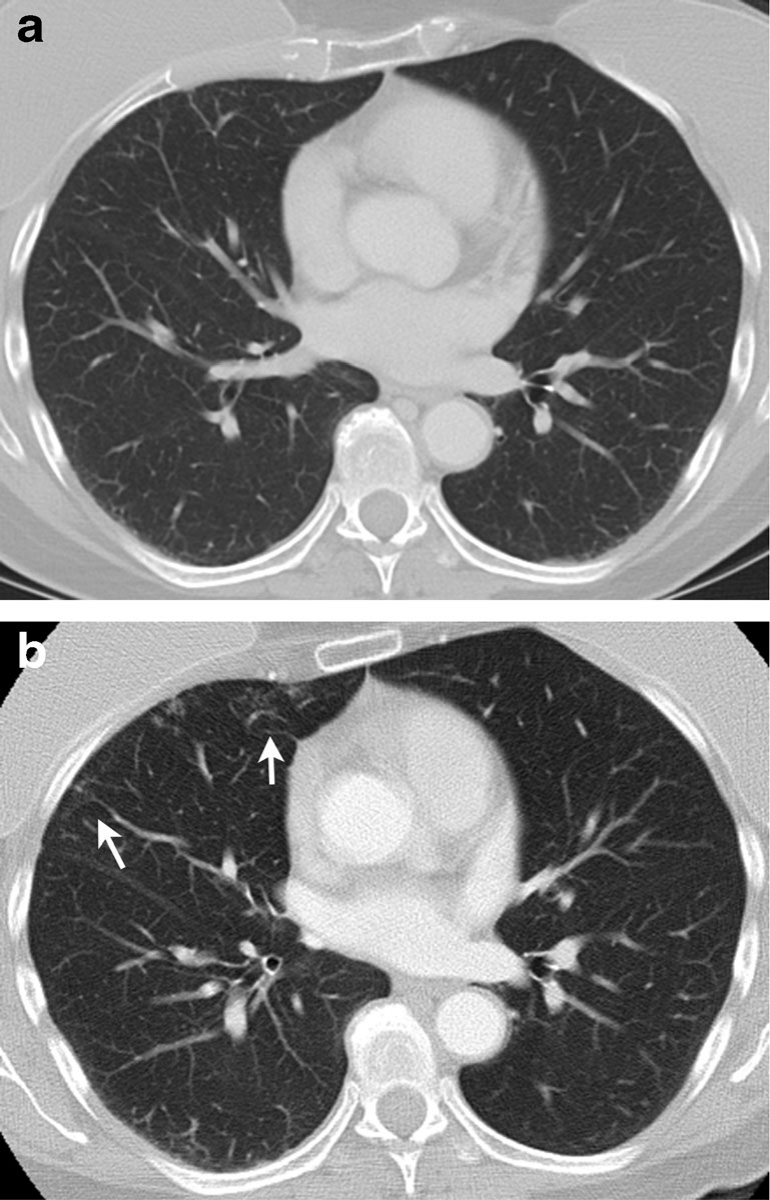
67-year-old woman with metastatic HER2/neu-positive breast cancer being treated with trastuzumab. Patient had prior radiotherapy in right breast. Axial contrast-enhanced lung window CT image before (**a**) and after (**b**) treatment with trastuzumab shows interval appearance of peripheral ground-glass opacities in right middle lobe (*arrows* in **b**) in radiation field suggestive of radiation recall pneumonitis

MTTs have class-specific and drug-specific toxicities. m-TOR inhibitors such as everolimus are associated with drug-induced pneumonitis manifesting radiologically as ground-glass and consolidative opacities [48]. Radiation recall pneumonitis refers to an inflammatory reaction in the lungs within the previously treated radiation field after the start of chemotherapy, and can be seen occasionally with HER2 inhibitors (Fig. 12) [60]. Trastuzumab has been associated with cardiotoxicity, especially when administered with or after doxorubicin. The incidence of cardiac dysfunction in patients receiving trastuzumab ranges from 2 to 28 % [61]. The most common manifestation of trastuzumab cardiotoxicity is an asymptomatic decrease in left ventricular ejection fraction (LVEF) [62]. In contrast to doxorubicin-associated cardiotoxicity, trastuzumab-induced cardiotoxicity is not related to cumulative dose and is often reversible with discontinuation of treatment [63]. Multiple-gated acquisition (MUGA) scan and echocardiogram are used for determination of LVEF in breast cancer patients before and after chemotherapy [64]. The biochemical marker troponin I is also useful for monitoring cardiotoxicity as well as for identifying patients who are at risk for cardiotoxicity and less likely to recover [65]. Treatment discontinuation is recommended in patients with a greater than 16 % drop in LVEF compared to baseline or a decline in LVEF to below the lower limit of normal, and in patients who develop symptomatic cardiac failure [66]. Adverse effects of lapatinib include fatigue, nausea, rash, diarrhoea, neutropenia, hepatotoxicity, interstitial lung disease and pneumonitis [67].

## Conclusions

Systemic treatment for breast cancer includes a combination of cytotoxic, hormonal and molecular-targeted therapies in various stages of treatment. The assessment of response to these agents on restaging scans has significant a impact on the patient’s treatment course. Imaging modalities such as CT, MRI, FDG-PET/CT and bone scintigraphy each have a distinct role in monitoring response to various treatment strategies. Familiarity with the response patterns on imaging and toxicities associated with these therapies can enhance the key role played by radiologists in patient management.
